# Dynamics of Glycemic Status and Glucose Metabolism Markers 12 Months After Coronary Artery Bypass Grafting and Their Relationship with the Annual Prognosis of Patients

**DOI:** 10.3390/jcm14020351

**Published:** 2025-01-08

**Authors:** Alexey N. Sumin, Natalia A. Bezdenezhnykh, Ekaterina V. Belik, Yulia A. Dyleva, Andrey V. Bezdenezhnykh, Olga V. Gruzdeva, Olga L. Barbarash

**Affiliations:** 1 Federal State Budgetary Scientific Institution, Research Institute for Complex Issues of Cardiovascular Diseases, Academician LS Barbarash Boulevard, 6, Kemerovo 650002, Russia; n_bez@mail.ru (N.A.B.); sionina.ev@mail.ru (E.V.B.); dyleva87@yandex.ru (Y.A.D.); o_gruzdeva@mail.ru (O.V.G.); olb61@mail.ru (O.L.B.); 2 Limited Liability Company, Family Health and Reproduction Center Krasnaya Gorka, Suvorova st., 3A, Kemerovo 650044, Russia; andrew22014@mail.ru; 3 Federal State Budgetary Educational Institution of Higher Education, Kemerovo State Medical University, Voroshilova st., 22A, Kemerovo 650056, Russia

**Keywords:** coronary heart disease, coronary artery bypass surgery, carbohydrate metabolism disorders, diabetes mellitus, one-year prognosis, glucose, glycated hemoglobin, fructosamine, 1,5-anhydroglucitol

## Abstract

**Background and Objectives:** We aim to evaluate the dynamics of glycemic status and markers of carbohydrate metabolism 12 months after coronary artery bypass grafting (CABG) and their relationship with the one-year prognosis. **Materials and Methods:** The analysis of outcomes of 653 patients during 1 year after coronary artery bypass grafting is presented. In those patients who visited the study center after 1 year, markers of carbohydrate metabolism (glucose, glycated hemoglobin, fructosamine, 1.5 anhydroglucitol) were assessed; in 371 of them, they were studied at three points—before surgery, before discharge from the hospital, and one year after surgery. The influence of these indicators on the incidence of cardiovascular events (death from any cause, myocardial infarction, stroke, repeat myocardial revascularization, surgical interventions on non-coronary arteries, amputations due to peripheral atherosclerosis, emergency hospitalizations due to cardiovascular disease, or combined endpoint [CEP]) was assessed during the year after CABG. Groups with (*n* = 59)/absence (*n* = 594) of the combined endpoint were formed and compared based on the dynamics of carbohydrate metabolism markers over the course of a year. Additionally, factors associated with the development of major adverse cardiovascular events (MACE) after CABG were assessed. **Results:** After 1 year, the number of patients with type 2 diabetes increased from 23.9% to 25.6% and prediabetes from 17.2% to 26.6% (*p* < 0.001). Among patients with diabetes mellitus, the following dynamics of carbohydrate metabolism markers were noted: a decrease in glucose levels in both groups (with or without CEP), glycated hemoglobin in the group without CEP, and fructosamine in the group with CEP. There were no differences in the intergroup comparison of all the described markers (glucose, fructosamine, glycated hemoglobin) and carbohydrate metabolism at all points. The following factors were associated with the development of MACE within a year after CABG: the presence of peripheral arterial disease, preoperative fibrinogen level, the risk of surgery according to the EuroSCORE scale, and off-pump CABG. **Conclusions:** In patients with diabetes mellitus one year after coronary artery bypass grafting, a decrease in glucose and glycated hemoglobin levels was noted. No differences in the dynamics of carbohydrate metabolism markers were found in the groups of patients with and without cardiovascular complications. The impact of glycated hemoglobin dynamics one year after CABG on long-term prognosis requires further research.

## 1. Introduction

In patients with diabetes mellitus and coronary artery disease, the choice of a myocardial revascularization strategy is often made in favor of coronary artery bypass grafting (CABG) [1]. This treatment tactic is due to the more frequent presence of multivessel coronary artery disease, as well as the shorter duration of coronary stents functioning in diabetes mellitus [2]. The attention of researchers during coronary artery bypass grafting in the presence of concomitant diabetes mellitus is mainly aimed at determining the optimal parameters of glycemic status before the planned operation [3], the correction of glucose levels in the perioperative period [4,5], the identification of possible additional disorders of carbohydrate metabolism before surgery [6,7], and the influence of these parameters on the immediate results of coronary artery bypass grafting. Currently, it has been shown that in patients with diabetes, strict glucose control (<7.8 mmol/L) and relatively large glucose fluctuations (≥4.4 mmol/L) were independently associated with adverse in-hospital outcomes [8]. At the same time, a number of studies failed to identify an association between preoperative glycated hemoglobin (HbA1c) levels and in-hospital mortality after CABG [9]. It was also shown that elevated preoperative HbA1c had no prognostic value for early complications and intermediate postoperative outcomes [10]. Also, studies by our group demonstrated that preoperative levels of an alternative marker of carbohydrate metabolism were one of the independent predictors of the development of in-hospital complications after CABG in patients with diabetes [11,12].

Less attention has been paid to the impact of preoperative carbohydrate metabolism disorders on long-term outcomes after CABG [3,9,13,14]. Thus, in the study by Robich MP et al. [9], it was shown that higher HbA1c levels before surgery are associated with worse long-term survival at five-year follow up. Similar results were obtained in a later study based on the results of a 10-year follow up of patients with diabetes after CABG [13].

At the same time, there is practically no information on the dynamics of various indicators of carbohydrate metabolism after CABG surgery, although such dynamics can potentially additionally affect the long-term results of CABG [15]. Therefore, we conducted the present study, the purpose of which was to evaluate the dynamics of glycemic status and markers of carbohydrate metabolism 12 months after coronary artery bypass grafting and their relationship with the one-year prognosis.

## 2. Methods

### 2.1. Study Population

From 22 March 2011 to 22 March 2012, a registry study called the “Coronary Artery Bypass Grafting (CABG) Registry” was conducted at the Research Institute for Complex Issues of Cardiovascular Diseases. The principle of patient selection for this registry is described in detail in previous articles written using the same patient sample [7,16].

CABG was performed in 708 patients, included in the main registry study. Upon admission to the hospital to prepare for CABG, all patients underwent a glycemic status examination. Perioperative management, including diagnosis of carbohydrate metabolism disorders before coronary artery bypass grafting and patient examination, is described in detail in previous articles written using the same patient sample [7,16]. Perioperative glycemic management was carried out in accordance with the national and international recommendations that were current at that time [17,18].

**Figure 1:**
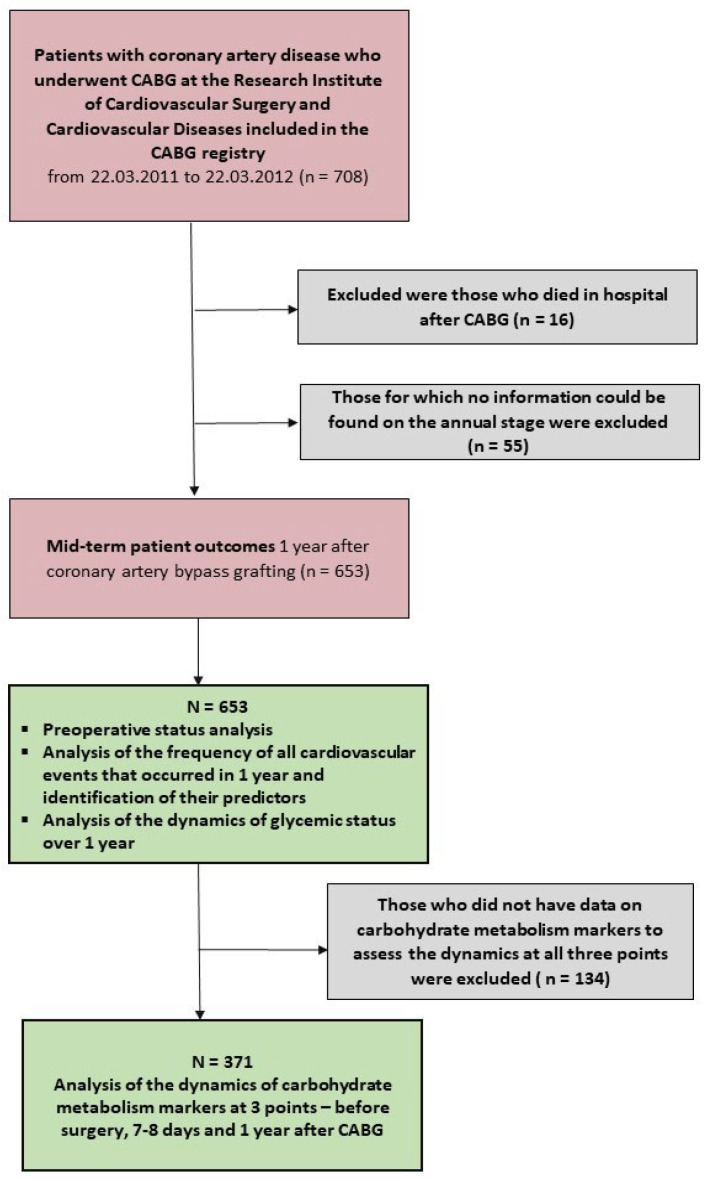
Flowchart of the study design. Notes: CABG—coronary artery bypass grafting.

The sample of patients used for further study included 708 patients who underwent CABG from 2011 to 2012 (Figure 1). Patients who died in the hospital during the index hospitalization after CABG were excluded from this analysis (*n* = 16). Patients were invited by phone to visit the study center 1 year after the index coronary artery bypass grafting to collect information and for examination. All patients who visited the study center signed informed consent for the examination, anonymized processing, and use of data. The study protocol was approved by the Local Ethics Committee of the Research Institute (Protocol No. 20110907, date of approval: 7 September 2011).

### 2.2. Data Collection

The scope of examinations conducted after 1 year included blood sampling, a study of routine blood biochemistry parameters and additional parameters of carbohydrate metabolism in dynamics, echocardiography, and duplex ultrasound of the carotid arteries. Also, all patients had their blood glucose and glycated hemoglobin determined upon a follow-up call, and if the results were insufficient to establish a diagnosis of carbohydrate metabolism disorders (CMD), an outpatient oral glucose tolerance test was recommended. A follow-up examination by an endocrinologist was conducted, and a diagnosis of CMD was established after additional examination. If the patient was unable to visit the study center, all possible information on remote outcomes was collected by telephone (contact with the patient or his relative). In the absence of telephone contact with the patient, the team of research doctors acted in several stages. The first stage was an audit of all these patients and the collection of information from the corporate medical portal on all visits to the clinic and hospitalizations and patient deaths during this study. At this stage, it was possible to obtain information on a significant proportion of deceased patients since the coronary artery bypass grafting registry located on the corporate portal contains regularly updated information from the civil registry offices. Letters with contact details of the study physician and a request to come for an examination were sent to all remaining addresses. As a result, information satisfactory for processing was received from 653 patients. Of these, 505 visited the study center in person (77.3%), and information was received from 148 patients (22.6%) by phone or in the manner described above.

Glucose, glycated hemoglobin (HbA1c), fructosamine, and 1.5 anhydroglucitol were determined in all 505 patients who came for a personal visit. In this case, the dynamics of these markers were only analyzed among those patients who had data on all three points of the specified markers: before coronary artery bypass grafting, on the 7–8th day after CABG, and after 1 year—there were 371 such patients. At the same time, glucose, which is routinely determined in 100% of patients at all three points, was analyzed among all 505 patients who came for a visit after 1 year.

### 2.3. Follow-Up Measures

The occurrence of the combined endpoint (CEP) was considered the occurrence of at least one of the outcomes listed above within 1 year after coronary artery bypass grafting. The following were considered as all being cardiovascular events after 1 year: death from any cause, myocardial infarction, stroke, repeat myocardial revascularization, surgical interventions on non-coronary arteries, amputations due to peripheral atherosclerosis, and emergency hospitalizations due to cardiovascular disease. The following were taken into account as major adverse cardiovascular events (MACE): death from any cause, myocardial infarction, and stroke. All cerebral strokes and myocardial infarctions that occurred during the year of observation were taken into account, including non-fatal postoperative ones. For further analysis, patients were divided into 2 groups: group 1 consisted of 59 patients who had at least one event related to MACE, and the second group consisted of 594 patients who did not have any events related to MACE after a year.

### 2.4. Statistical Analyses

Statistical processing was performed using the standard software packages “STATISTICA 8.0” (Dell Software, Inc., Round Rock, TX, USA) and SPSS 17.0 (IBM, Armonk, NY, USA). The normality of the distribution of quantitative data was checked using the Shapiro–Wilk criterion. Since the distribution of all quantitative features differed from normal, they are presented as a median and quartiles (25th and 75th percentiles). To compare three groups by quantitative features, the Kruskal–Wallis criterion was used, and for qualitative ones, the chi-square was used. For pairwise comparison of groups, the Mann–Whitney test and χ2 (chi-square) were used. For a small number of observations, the Fisher exact test with Yates correction was used. Logistic regression analysis was used to identify predictors of adverse outcomes. The level of critical significance (*p*) in the regression analysis was taken to be 0.05.

## 3. Results

**Figure 2:**
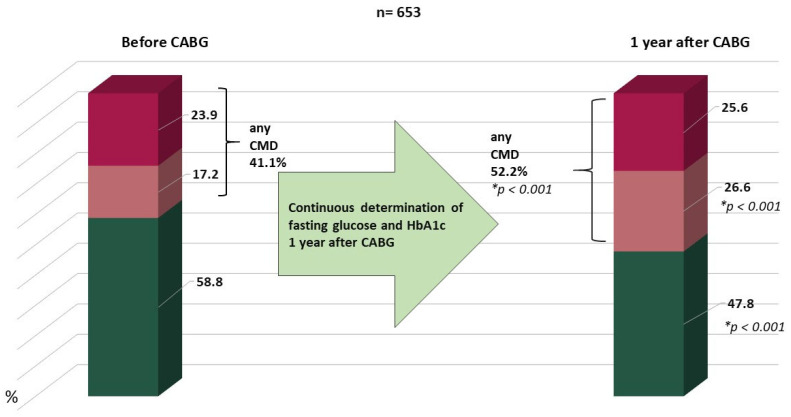
Increase in the proportion of carbohydrate metabolism disorders over 1 year. Notes: CABG—coronary artery bypass grafting, CMD—carbohydrate metabolism disorders, HbA1c—glycated hemoglobin, IFG—impaired fasting glucose, IGT—impaired glucose tolerance, * compared to preoperative values.

The overall dynamics of carbohydrate metabolism are shown in Figure 2. After 1 year, the number of patients with type 2 diabetes increased from 23.9 to 25.6% and prediabetes from 17.2 to 26.6%.

**Figure 3:**
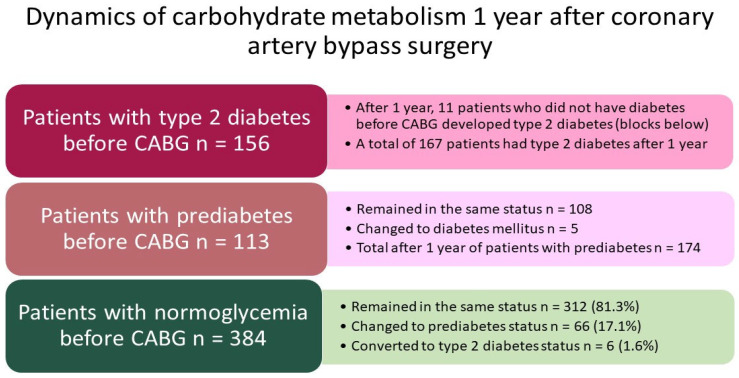
Detailed analysis of changes in glycemic status within 1 year after coronary artery bypass surgery. Notes: CABG—coronary artery bypass grafting.

The detailed dynamics of carbohydrate metabolism for 1 year is shown in Figure 3. Before CABG, 156 (23.9%) of these 653 patients had type 2 diabetes, 112 (17.2%) had prediabetes (impaired fasting glucose, impaired glucose tolerance, or a combination of both), and 384 (58.8%) patients had normoglycemia. The proportion of diabetes increased by 1.7% over the year (*p* = 0.580), and the proportion of prediabetes increased by 9.4% over the year (*p* < 0.001). Eleven patients switched from the normoglycemia group to the group of one or another carbohydrate metabolism disorder. Before CABG, 112 patients had prediabetes, and after a year, there were 108 such patients, and 5 patients switched to the status of type 2 diabetes.

The dynamics of carbohydrate metabolism in the normoglycemia group were as follows: before CABG, there were 384 such patients (100%), and 312 patients remained in this status, 66 (16.9%) patients developed prediabetes, and 6 (1.6% of 384) developed type 2 diabetes. In total, over 1 year, 11 patients (1.7%) switched to the status of diabetes in this group. In 66 patients out of 384 who did not have CMD, prediabetes was detected during the observation period, and impaired fasting glycemia or impaired glucose tolerance led to the development of type 2 diabetes in 6 patients (1.6% of 384). 

The anamnestic and clinical characteristics of patients before coronary artery bypass grafting are described in Table 1. Patients in the groups with and without CEP did not differ in gender and age, the presence of arterial hypertension, diabetes mellitus and prediabetes, smoking status, FC CHF, or angina.

At the same time, patients who had suffered CVD events were less likely to engage in mental work and more often in physical work (*p* < 0.001) and were less likely to have higher education (*p* = 0.027). Also, obesity was detected less often in the CEP group (*p* = 0.044); apparently, the obesity paradox was manifested here. Patients who underwent CEP more often had intermittent claudication before CABG (*p* < 0.001) and a shorter pain-free walking distance (*p* = 0.007). There was a tendency towards a higher incidence of strokes suffered before CABG in group 1, which did not reach statistical significance (*p* = 0.061). Patients of the first group more often had interventions on the carotid arteries before CABG (*p* = 0.037), and a significantly higher risk was assessed by the EuroSCORE 2 scale (*p* < 0.001).

The cardiovascular events for 1 year after coronary artery bypass grafting are described in Table 2. A large percentage of patients came for the visit in person (77%). CEP occurred during 1 year of observation in 59 (9.0%) of all examined patients. Major adverse cardiovascular events occurred in 29 patients (4.4%), and death from any cause was registered during the year in 18 patients (2.6%).

The characteristics of coronary artery bypass grafting are shown in Table 3. There was a tendency towards less frequent use of CPB and more frequent off-pump surgery in group 1, which did not reach significance (*p* = 0.053). At the same time, patients with CEP had significantly more frequent combined surgery (17.1 and 8.1%, respectively, *p* = 0.022), mostly due to operations on the aortic valve (*p* = 0.037). Patients in both groups were comparable in terms of CPB duration, total duration of surgery and aortic clamping, and intraoperative blood loss.

Patients in the CEP group received aspirin pre-hospital less often; otherwise, the groups did not differ in terms of the main drug therapy before CABG (Table 4). Patients with CEP, when compared between groups with patients without adverse events, at the 1-year stage were significantly less likely to take aspirin (*p* < 0.001), beta blockers (*p* = 0.032), angiotensin-converting enzyme inhibitors (*p* = 0.013), and statins (*p* = 0.035). When comparing the pre-hospital and 1-year stages, patients in each group had a lower percentage of taking aspirin, beta blockers, and angiotensin-converting enzyme inhibitors (*p* < 0.05), but not mineralocorticoid antagonists, diuretics, and calcium antagonists. As for antihyperglycemic therapy, patients with diabetes mellitus received it at the 1-year stage with the same frequency as at the hospital stage in both groups. Moreover, both groups at the annual stage had a higher median average daily insulin dose (Table 4). 

**Figure 4:**
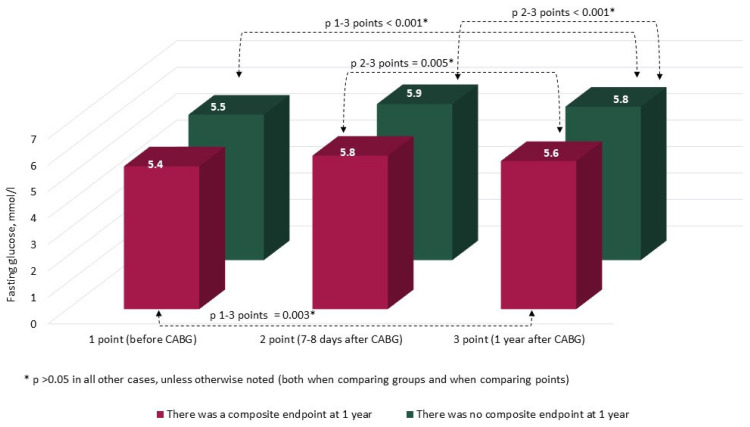
Dynamics of fasting glucose in groups over 1 year (*n* = 371). Notes: CABG—coronary artery bypass grafting. * *p* > 0.05.

**Figure 5:**
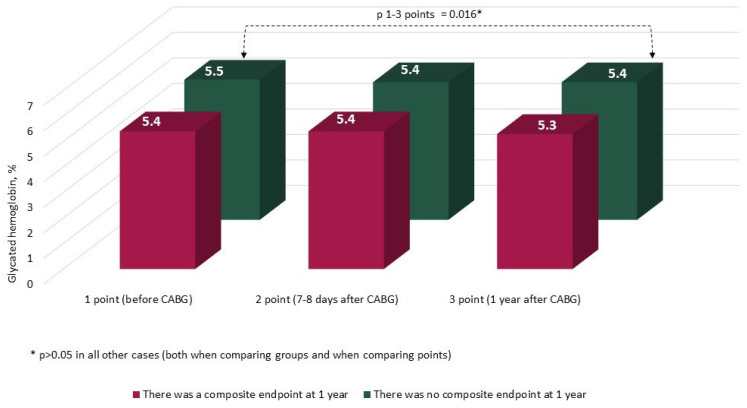
Dynamics of glycated hemoglobin (HbA1c) in groups over 1 year (*n* = 371). Notes: CABG—coronary artery bypass grafting. * *p* > 0.05.

**Figure 6:**
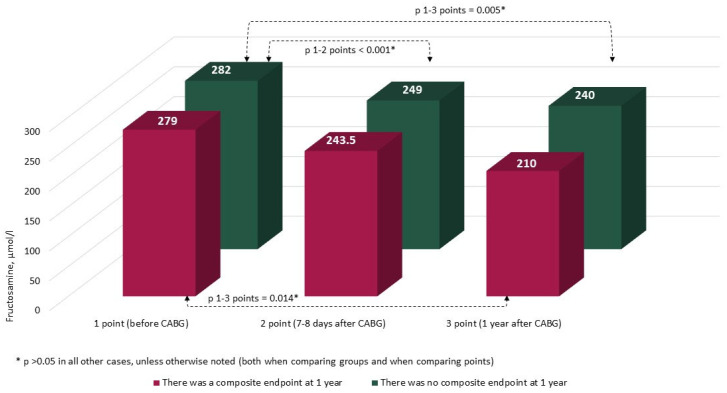
Dynamics of fructosamine in groups over 1 year (*n* = 371). Notes: CABG—coronary artery bypass grafting. * *p* > 0.05.

**Figure 7:**
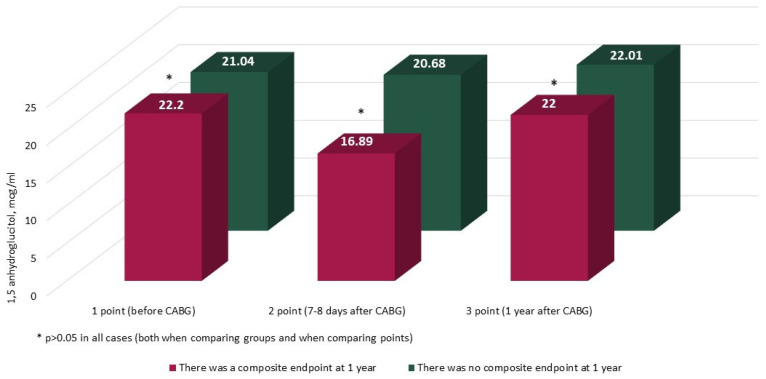
Dynamics of 1.5 anhydroglucitol in groups over 1 year (*n* = 371). Notes: CABG—coronary artery bypass grafting. * *p* > 0.05.

The characteristics and annual dynamics of carbohydrate metabolism markers are shown in Figure 4, Figure 5, Figure 6 and Figure 7. In both groups, the glucose level was significantly lower after 1 year in comparison with the preoperative and postoperative levels (*p* in all cases < 0.017) (Figure 4). Glycated hemoglobin in the group without CEP was lower than before CABG, and in the group with CEP, the levels of glycated hemoglobin did not differ (Figure 5). Fructosamine in the group with CEP was significantly lower after 1 year in comparison with the preoperative level (1 point, *p* = 0.015) (Figure 6). In the intergroup comparison of all the described markers (glucose, fructosamine, glycated hemoglobin) and carbohydrate metabolism at all points, there were no differences (*p* > 0.05). The level of 1.5 anhydroglucitol did not differ either in the intergroup comparison at each point or in the comparison of annual dynamics (Figure 7).

The data of preoperative instrumental and routine laboratory examinations are presented in Table 5. Patients in the two groups did not differ in the severity of coronary artery disease. At the same time, multifocal atherosclerosis (damage to two or more arterial beds) was significantly more often detected before CABG in those patients who subsequently underwent unfavorable CEP (54.2 and 44.8% in the two groups, respectively, *p* < 0.001). At the same time, damage to three vascular beds was found in 35.6% of patients in group 1 compared to 14.7% of patients in group 2 (*p* < 0.001). Patients in group 1 had a greater thickness of the intima–media complex (*p* = 0.043). According to echocardiography, there were no differences in EF, linear, and volumetric indicators of the left ventricle. At the same time, patients differed in diastolic parameters; there were lower E/A (early to late diastolic transmitral flow ratio, *p* = 0.039) and DT (deceleration time, *p* = 0.004) parameters. At the same time, IRT (isovolumic relaxation time) was higher in the CEP group, but the trend did not reach statistical significance (*p* = 0.059). In terms of other echocardiography parameters, the groups were comparable. When analyzing routine laboratory data, attention is drawn to the higher medians of preoperative values of soluble fibrin monomer complexes and fibrinogen in the group undergoing CEP (Table 5, *p* < 0.001 and *p* = 0.004, respectively).

When analyzing routine laboratory data, attention is drawn to the higher medians of preoperative values of soluble fibrin monomer complexes and fibrinogen in the group undergoing CEP (*p* < 0.001 and *p* = 0.004, respectively).

In the binary logistic regression model (Table 6, forward LR method), no carbohydrate metabolism markers were associated with either the development of MACE or the development of the combined endpoint (including all cardiovascular events) within a year after CABG. We identified the following independent factors associated with the development of MACE: the presence of peripheral arterial disease (B = 0.949, *p* = 0.030), preoperative fibrinogen level (B = 0.276, *p* = 0.017), the risk of surgery according to the EuroSCORE scale (B = 0.173, *p* = 0.004), and off-pump CABG (*p* = 0.029) (Table 6). For this model, the statistical significance was χ^2^(2) = 20.048, *p* = 0.001, the Nagelkerke R2 value was 0.108, and the model correctly classified 95.0% of cases (Supplementary Tables S1–S3).

## 4. Discussion

This study shows that one year after coronary artery bypass grafting, an increase in the number of patients with carbohydrate metabolism disorders was noted. Among patients with diabetes mellitus, the following dynamics of carbohydrate metabolism markers were noted: a decrease in glucose levels in both groups, glycated hemoglobin in the group without CEP, and fructosamine in the group with CEP. This decrease may be explained by better glycemic control in patients after successful surgical treatment of coronary heart disease, as well as by improved compliance of such patients with regard to lifestyle and medication therapy. There were no differences in the intergroup comparison of all the described markers (glucose, fructosamine, glycated hemoglobin) and carbohydrate metabolism at all points. The level of 1.5 anhydroglucitol did not differ either in the intergroup comparison at each point or in the comparison of annual dynamics. This is probably explained by the fact that its diagnostic sensitivity, 1,5-AG, may be insufficient in individuals with a slight increase in glucose levels, which is present in prediabetes, especially in the case of the predominance of fasting hyperglycemia. When analyzing the factors associated with the annual prognosis, it was not possible to identify an association with CEP or MACE of carbohydrate metabolism markers at any measurement points.

Previous studies have noted the effect of preoperative glycated hemoglobin levels on long-term results after CABG in patients with diabetes. This has been shown when evaluating the results of CABG after 5 years or more [13,19], after 3.5 years [3], and after 2.6 years [9]. For example, it has been shown that the risk of death increases by 13% for each unit increase in HbA1c (OR 1.13; 95% CI 1.07–1.19; *p* < 0.001) [9]). At the same time, the level of glycated hemoglobin had a less pronounced effect on the immediate results of CABG. Only a few studies have noted the effect of preoperative carbohydrate metabolism disorders on in-hospital outcomes [12,20]. For example, in the study by Liu et al. [20], a higher preoperative HbA1c level was associated with a longer postoperative stay in the ICU and a higher risk of acute renal function damage in patients with diabetes after CABG. Thus, after adjusting for patient risk, higher HbA1c values were not associated with higher in-hospital mortality or morbidity rates [9]. In another recent study, elevated preoperative HbA1c had no prognostic value for early complications and intermediate postoperative outcomes [10]. Similar data were obtained for patients without diabetes mellitus; elevated preoperative HbA1c may be associated with an increased risk of complications, but this association is mediated by the presence of comorbidities associated with hyperglycemia [6]. Based on these results, it was suggested to not strive to achieve a reduction in elevated glycated hemoglobin levels in the preoperative period and to delay CABG because of this [10].

To understand the differences between the short-term and long-term prognostic value of preoperative glycated hemoglobin levels, the results of a post hoc subgroup analysis of the DACAB (Different Antiplatelet Therapy Strategy After Coronary Artery Bypass Graft Surgery) study are of interest. This analysis examined the effect of baseline HbA1c levels on the status of the vein bypass graft one year after coronary artery bypass grafting [21]. When studying the subgroups of patients with preoperative HbA1c levels <6.5% compared with HbA1c levels ≥6.5%, it turned out that lower baseline HbA1c levels were associated with higher vein bypass graft patency 1 year after CABG [21]. Apparently, the results of our study show that the adverse effect of poorly compensated carbohydrate metabolism disorders on the patency of bypasses has not yet had time to be realized in the effect on the one-year prognosis after CABG; this requires a longer observation period.

Since glycemic control before hospitalization, assessed by HbA1c, is a predictor of long-term survival, a natural question arises—is it possible to change this risk by improving glycemic control after surgery? In this regard, the results of the study by Turgeon et al. [3], who studied long-term outcomes after CABG taking into account the HbA1c values achieved in the postoperative period, are interesting. It was shown that the most optimal HbA1c levels were within 6.1–7.0%, which was associated with a lower risk of MACE and unstable angina compared to achieving an HbA1c level >8.0% and a lower risk of death compared to achieving an HbA1c level ≤6.0%. In this study, we were able to show that some carbohydrate metabolism indicators decrease when assessed one year after surgery, but no associations with the one-year prognosis were found. Whether these dynamics of carbohydrate metabolism markers will affect the long-term results after surgery should be studied in further studies. When conducting such studies, it will be necessary to take into account not only the degree of compensation/decompensation of diabetes mellitus but also other drug therapies. Thus, it was shown that among patients with diabetes mellitus, blood pressure control and statin therapy were the most important perioperative cardiometabolic determinants of survival at 5 years after CABG [14]. It is quite possible that the adequacy of not only preoperative but also postoperative therapy for comorbid diseases may have a significant impact on the long-term prognosis in patients with diabetes mellitus after CABG.

When evaluating the results of this study, it is necessary to take into account its limitations. First, this study was a retrospective analysis, which could lead to bias in the results (for example, we did not include patients who died before the examination one year later). Second, this study was conducted in one center, which limits the possibility of extending its results to the general population of patients with diabetes mellitus after CABG. Third, the follow-up period for patients after CABG was limited to one year, which did not allow us to identify the prognostic value of the dynamics of carbohydrate metabolism markers after CABG. To overcome this limitation, it is planned to evaluate the long-term results in this cohort of patients.

## 5. Conclusions

One year after CABG, an increase in the number of patients with carbohydrate metabolism disorders was noted, primarily due to patients with prediabetes. At the same time, in patients with diabetes mellitus one year after coronary artery bypass grafting, a decrease in glucose and glycated hemoglobin levels was noted. No differences in the dynamics of carbohydrate metabolism markers were found in the groups of patients with and without cardiovascular complications. The impact of glycated hemoglobin dynamics one year after CABG on long-term prognosis requires further research.

## Figures and Tables

**Table 1:** Anamnestic and clinical characteristics of patients before coronary artery bypass grafting (*n* = 653).

Indicator	Group With CEP at 1 Year (*n* = 59)	Group Without CEP at 1 Year (*n* = 594)	*p*
Men (n,%)	51 (86.4)	467 (78.6)	0.157
Women (n, %)	8 (13.6)	127 (21.4)	0.157
Age (years, Me [LQ; UQ])	59.0 (54.0; 65.5)	58.0 (54.1; 64.0)	0.561
Mental work (n, %)	12 (20.3)	209 (35.1)	<0.001
Physical labor (n, %)	47 (79.7)	385 (64.8)	<0.001
Higher education (n, %)	6 (10.2)	134 (22.5)	0.027
BMI (kg/m^2^, Me [LQ;UQ])	27.2 (24.2; 29.6)	28.0 (25.2; 31.19)	0.086
Obesity (n, %)	14 (23.7)	208 (35.0)	0.044
Type 2 diabetes mellitus before CABG (n, %)	10 (20.3)	146 (24.6)	0.524
Prediabetes before CABG (IFG or IGT) (n, %)	12 (18.6)	101 (17.0)	0.646
Any disturbance of carbohydrate metabolism before CABG (n, %)	19 (32.2)	226 (38.1)	0.472
Arterial hypertension (n, %)	55 (93.2)	521 (87.7)	0.210
III—IV FC angina (n, %)	24 (40.7)	238 (40.1)	0.927
III-IV NYHA FC heart failure (n, %)	16 (27.1)	173 (29.1)	0.746
Smoking at the time of CABG (n, %)	25 (42.4)	205 (34.5)	0.228
Intermittent claudication (n, %)	16 (27.1)	67 (11.3)	<0.001
Pain-free walking distance (m, Me [LQ; UQ])	150 (70.0; 300.0)	450.0 (200.0; 1000.0)	0.007
History of myocardial infarction (n, %)	38 (64.4)	383 (64.5)	0.936
History of stroke (n, %)	8 (13.5)	40 (6.7)	0.061
History of PCI (n, %)	6 (10.2)	51 (8.6)	0.711
History of CABG (n, %)	0 (0)	5 (0.8)	0.682
History of surgery on carotid arteries (n, %)	4 (6.8)	13 (2.2)	0.037
History of surgery on arteries of the lower extremities (n, %)	1 (1.7)	3 (0.5)	0.271
History of thyroid disease (n, %)	2 (3.4)	34 (5.7)	0.439
EuroSCORE 2 (%, Me [LQ; UQ])	2.4 (1.7; 4.1)	1.7 (1.1; 2.5)	<0.001

Notes: CEP—combined endpoint, Me [LQ; UQ]—median with upper and lower quartile, BMI—body mass index, FC—functional class, NYHA—New York Heart Association, PCI—percutaneous coronary intervention, CABG—coronary artery bypass grafting, IFG—impaired fasting glucose, IGT—impaired glucose tolerance, EuroSCORE—European System for Cardiac Operative Risk Evaluation.

**Table 2:** Major cardiovascular events within 1 year after coronary artery bypass grafting (*n* = 653).

Indicator	*n*, %
Visit to the study center after 1 year	505 (77.3)
Telephone contact or other means of communication after 1 year, except for a visit	148 (22.7)
All cardiovascular events at 1 year (combined endpoint)	59 (9.0)
Major adverse cardiovascular events in 1 year	29 (4.4)
Death from all causes in 1 year	18 (2.6)
Myocardial infarction	4 (0.06)
Stroke	7 (1.1)
Percutaneous coronary intervention	6 (0.1)
Carotid artery intervention	10 (1.5)
Intervention in the arteries of the lower extremities	12 (1.8)
Recurrence of angina	6 (0.1)
Emergency hospitalizations for CVD	18 (2.7)

Notes: CVD—cardiovascular disease.

**Table 3:** Characteristics of coronary artery bypass grafting (*n* = 653).

Indicator	Group with CEP at 1 Year (*n* = 59)	Group Without CEP at 1 Year (*n* = 594)	*p*
On-pump heart surgery (n, %)	47 (79.6)	525 (88.4)	0.053
Cardiopulmonary bypass laminar mode (n, % of the number of patients on pump)	37 (78.7)	431 (82.1)	0.600
Cardiopulmonary bypass pulse mode (n, % of the number of patients on pump)	10 (21.2)	94 (17.9)	0.600
Off-pump heart surgery (n, %)	12 (20.3)	69 (11.6)	0.053
Isolated CABG (n, %)	49 (83.0)	546 (91.9)	0.022
Combined operations (n, %)	10 (17.0)	48 (8.1)	0.022
Carotid endarterectomy (n, %)	2 (3.4)	9 (1.5)	0.296
Ventriculoplasty (n, %)	4 (6.7)	26 (4.4)	0.401
Radiofrequency ablation (n, %)	1 (1.7)	16 (2.7)	0.633
Mitral valve (n, %)	1 (1.7)	2 (0.3)	0.146
Aortic valve (n, %)	2 (3.4)	4 (0.7)	0.037
Thrombectomy (n, %)	3 (5.1)	14 (2.4)	0.220
Total duration of surgery (min, Me [LQ; UQ])	246.0 [192.0; 282.0]	246.0 [204.0; 300.0]	0.472
Cardiopulmonary bypass duration (min, Me [LQ; UQ])	98.0 [78.0; 125.0]	95 [78.0; 110.0]	0.207
Duration of aortic clamping (min, Me [LQ; UQ])	65.0 [52.0; 77.0]	60.0 [50.0; 73.0]	0.215

Notes: CEP—combined endpoint, Me (LQ; UQ)—median with upper and lower quartile, CABG—coronary artery bypass grafting.

**Table 4:** Medicament therapy in groups with/without CEP (*n* = 653).

Indicator (n, %, Unless Otherwise Stated)		Group with CEP at 1 Year (*n* = 59)		Group Without CEP at 1 Year (*n* = 594)		*p* #
Acetylsalicylic acid (n, %)	preoperatively	32 (54.2)		374 (63.0)		0.225
	pre-hospital	28 (47.4)	*p* * < 0.001	394 (66.3)	*p* * < 0.001	0.007
	in 1 year	14 (23.7)		286 (48.1)		<0.001
β blockers (n, %)	preoperatively	58 (98.3)		564 (94.9)		0.309
	pre-hospital	32 (54.2)	*p* * < 0.001	374 (63.0)	*p* * < 0.001	0.225
	in 1 year	15 (25.4)		284 (47.8)		0.032
Angiotensin-converting enzyme inhibitors (n, %)	preoperatively	51 (86.4)		506 (85.2)		0.928
	pre-hospital	31 (52.5)	*p* * = 0.001	273 (45.9)	*p* * = 0.005	0.290
	in 1 year	12 (20.3)		214 (36.0)		0.013
Angiotensin 2 receptor antagonists (n, %)	preoperatively	1 (1.7)		28 (4.7)		0.283
	pre-hospital	1 (1.7)	*p* * = 0.917	42 (7.0)	*p* * = 0.883	0.154
	in 1 year	1 (1.7)		43 (7.2)		0.488
Statins (n, %)	preoperatively	40 (67.8)		443 (74.6)		0.227
	pre-hospital	27 (45.8)	*p* * = 0.021	279 (47.0)	*p* * = 0.861	0.835
	in 1 year	15 (25.4)		282 (47.5)		0.032
Calcium channel blockers (n, %)	preoperatively	30 (50.9)		368 (62.0)		0.095
	pre-hospital	13 (22.0)	*p* * = 0.674	135 (22.7)	*p* * = 0.834	0.871
	in 1 year	11 (18.6)		132 (22.2)		0.981
Mineralocorticoid receptor antagonists (n, %)	preoperatively	15 (25.4)		95 (16.0)		0.171
	pre-hospital	5 (9.1)	*p* * = 0.751	51 (8.6)	*p* * = 0.454	0.839
	in 1 year	6 (10.7)		44 (7.4)		0.251
Thiazide-like diuretics (n, %)	preoperatively	3 (5.1)		57 (9.5)		0.308
	pre-hospital	6 (10.1)	*p* * = 0.298	32 (5.3)	*p* * = 0.101	0.434
	in 1 year	3 (5.1)		46 (7.7)		0.491
Loop diuretics (n, %)	preoperatively	4 (6.7)		39 (6.5)		0.831
	pre-hospital	1 (1.7)	*p* * = 0.558	12 (2.0)	*p* * = 0.667	0.905
	in 1 year	2 (3.4)		10 (1.8)		0.711
Insulin (n, %)	preoperatively	5 (8.5)		65 (10.9)		0.558
	pre-hospital	3 (5.0)	*p* * = 0.675	24 (4.0)	*p* * = 0.773	0.870
	in 1 year	3 (5.0)		26 (4.3)		0.365
Median insulin dose (U/day, Me [LQ; UQ])	preoperatively	28.0 (18.0; 36.0)		28.0 (18.0; 38.0)		0.733
	pre-hospital	34.0 (24.0; 44.0)	*p* * = 0.047	34.0 (20.0; 38.0)	*p* * = 0.018	0.733
	in 1 year	36.0 (22.0;46.0)		38.0 (26.0;48.0)		0.801
Oral medications for the treatment of diabetes mellitus (n,%)	pre-hospital	4 (5.1)	*p* * = 0.551	57 (9.6)	*p* * = 0.842	0.258
	in 1 year	5 (8.5)		55 (9.2)		0.586
Sulfonylurea drugs (n, %)	pre-hospital	3 (5.1)	*p* * = 0.674	32 (5.4)	*p* * = 0.794	0.900
	in 1 year	2 (3.4)		30 (5.0)		0.636
Metformin (n, %)	pre-hospital	1 (1.7)	*p* * = 0.611	25 (4.4)	*p* * = 0.754	0.260
	in 1 year	3 (5.1)		32 (5.4)		0.754
DPP-4 inhibitors (n, %)	pre-hospital	1 (1.7)	*p* * = 0.560	4 (0.7)	*p* * = 0.758	0.657
	in 1 year	2 (3.4)		6 (1.0)		0.562
GLP-1 agonists (n, %)	pre-hospital	1 (1.7)	*p* * = 0.999	2 (0.3)	*p* * = 0.654	0.789
	in 1 year	1 (1.7)		3 (0.5)		0.638

Notes: CEP—combined endpoint, CMD—carbohydrate metabolism disorders, *p* #—for intergroup comparison, *p* *—for comparison of pre-hospital and annual stages in each group, Me [LQ; UQ]—median with upper and lower quartile, DPP-4—dipeptidyl peptidase 4, GLP-1—glucagon-like peptide 1.

**Table 5:** Data of preoperative instrumental and routine laboratory examinations (*n* = 653).

Indicator	Group with CEP at 1 Year (*n* = 59)	Group Without CEP at 1 Year (*n* = 594)	*p*
Coronary angiography results (n, %)			
1 vessel disease	15 (25.4)	134 (22.6)	0.617
2 vessel diseases	13 (22.6)	162 (27.3)	0.386
3 vessel diseases	27 (45.8)	255 (42.9)	0.675
Stenosis of the left main coronary artery >50%	10 (16.9)	117 (19.7)	0.611
3 vessels and left main coronary artery	34 (6.7)	34 (5.7)	0.554
3 vessels or left main coronary artery	33 (55.9)	320 (53.9)	0.762
Aggregate results of instrumental examinations of non-coronary arterial basins (n, %, unless otherwise stated)			
Multifocal atherosclerosis, lesion of 3 arterial basins	21 (35.6)	87 (14.7)	<0.001
Multifocal atherosclerosis, lesion of 2 or more arterial basins	32 (54.2)	157 (44.8)	<0.001
Multifocal atherosclerosis, lesion of 2 arterial basins	25 (42.3)	199 (33.5)	0.171
Absence of multifocal atherosclerosis (damage to the coronary bed only)	13 (22.0)	308 (51.9)	<0.001
Carotid artery stenosis ≥50% before CABG	21 (16.2)	19 (12.1)	0.354
Stenosis of the arteries of the lower extremities before CABG	37 (28.7)	46 (29.0)	0.355
Intima-media thickness, mm (Me [LQ; UQ])	1.2 (1.0; 1.3)	1.1 (1.0; 1.2)	0.043
Echocardiography data, extended protocol (Me [LQ; UQ])			
LV end-diastolic volume (ml)	160.0 (133.0; 180.0)	155.0 (135.0; 188.0)	0.759
LV end-diastolic dimension (cm)	5.7 (5.3; 6.0)	5.6 (5.3 6.1)	0.692
LV end-systolic volume (ml)	66.0 (47.0; 104.0)	62 (50.0; 92)	0.816
LV end-systolic dimension (cm)	3.9 (3.5; 4.7)	3.8 (3.5; 4.6)	0.667
Left atrium (cm)	4.15 (3.8; 4.5)	4.2 (3.9; 4.5)	0.144
Interventricular septum (mm)	1.1 (1.0; 1.2)	1.1 (1.0; 1.2)	0.854
Posterior wall of the left ventricle (mm)	1.1 (1.0; 1.2)	1.1 (1.0; 1.2)	0.638
Right ventricle, cm	1.8 (1.75; 1.8)	1.8 (1.8; 1.9)	0.336
Right atrium, cm	4.3 (4.0; 4.6)	4.2 (3.8; 4.6)	0.374
Aorta, cm	3.4 (3.2; 3.7)	3.5 (3.3; 3.7)	0.334
LV ejection fraction, %	58.0 (46.0; 65.0)	60 (50.0; 64.0)	0.798
Pulmonary artery pressure, systolic, mmHg	12.0 (10.0; 30.0)	18 (12.0; 28.0)	0.257
E (cm/sec)	53.0 (46.0; 73.0)	61.0 (50.0; 75.0)	0.140
A (cm/sec)	68.0 (56.0; 88.0)	65.0 (53.0; 78.0)	0.438
E/A	0.7 (0.6; 1.2)	0.8 (0.7; 1.2)	0.039
DT, sec	190.0 (140.0; 204.0)	200.0 (180.0; 235.0)	0.004
IRT—isovolumic relaxation time, sec	111.0 (93.0; 122.0)	100.0 (90.0; 114.0)	0.059
Flow velocity (cm/sec)—Vf	59.0 (46.0; 61.0)	48.0 (42.0; 60.0)	0.254
LV myocardial mass according to Deveraux and Reichek, g	314.0 (246.3; 375.2)	306, 4 (250.4; 372.7)	0.395
LV myocardial mass index	166.1 (129.3; 198.6)	161.7 (134.2; 190.5)	0.181
Preoperative laboratory data (except carbohydrate metabolism) Me (LQ; UQ)			
Aspartate aminotransferase (IU/L)	23.0 (20.0; 31.0)	24.0 (19.0; 31.0)	0.626
Alanine aminotransferase (IU/L)	26.0 (17.0; 37.0)	25.0 (18.0; 36.9)	0.665
Total cholesterol (mmol/l)	5.0 (4.3; 6.0)	4.9 (4.2; 5.9)	0.691
Triglycerides (mmol/l)	1.8 (1.3; 2.3)	1.7 (1.3; 2.3)	0.393
HDL cholesterol (mmol/l)	0.97 (0.88; 1.15)	0.99 (0.83; 1.19)	0.937
LDL cholesterol (mmol/l)	3.02 (2.5; 4.1)	2.95 (2.3; 3.7)	0.290
Creatinine (µmol/l)	77.5 (65.0; 94.0)	76.0 (64.0; 91.0)	0.082
GFR CKD—EPI (ml/min/1.73 m^2^)	76.4 (62.9; 90.3)	82.4 (66.7; 102.6)	0.106
SFMCs (g/l)	7.5 (4.8; 12.0)	5.0 (4.0; 8.0)	<0.001
Fibrinogen (g/l)	5.3 (3.8; 6.2)	4.2 (3.5; 5.5)	0.004

Notes: CEP—combined endpoint, LV—left ventricle, E—early diastolic filling velocity of the LV, A—late diastolic filling velocity of the LV, E/A—the ratio of early and late diastolic transmitral flow, DT—deceleration time of early diastolic filling of transtricuspid blood flow, *IRT*—isovolumic relaxation time, Vf—flow velocity (cm/sec), HDL—high-density lipoprotein, LDL—low-density lipoprotein, GFR—glomerular filtration rate, CKD—EPI—Chronic Kidney Disease Epidemiology Collaboration, SFMCs—soluble fibrin monomer complexes.

**Table 6:** Binary logistic regression results (forward LR method): association of factors with the risk of developing MACE one year after coronary artery bypass grafting.

								95% CI.for EXP(B)	
		B	Lower	Upper	df	Sig.	Exp(B)	Lower	Upper
Step 1	EuroSCORE	0.183	0.059	9.650	1	0.002	1.201	1.070	1.348
	Constant	−3.441	0.275	156.543	1	0.000	0.032		
Step 2	Fibrinogen	0.243	0.113	4.590	1	0.032	1.275	1.021	1.591
	EuroSCORE	0.184	0.060	9.562	1	0.002	1.202	1.070	1.351
	Constant	−4.645	0.659	49.680	1	0.000	0.010		
Step 3	Fibrinogen	0.263	0.115	5.246	1	0.022	1.300	1.039	1.628
	EuroSCORE	0.183	0.060	9.415	1	0.002	1.201	1.069	1.351
	CABG on pump	−0.976	0.470	4.316	1	0.038	0.377	0.150	0.946
	Constant	−3.943	0.723	29.764	1	0.000	0.019		
Step 4	PAD	0.949	0.437	4.711	1	0.030	2.583	1.096	6.083
	Fibrinogen	0.276	0.116	5.676	1	0.017	1.317	1.050	1.653
	EuroSCORE	0.173	0.061	8.090	1	0.004	1.189	1.055	1.340
	CABG on pump	−1.038	0.476	4.764	1	0.029	0.354	0.139	0.900
	Constant	−5.102	0.930	30.088	1	0.000	0.006		

Notes: EuroSCORE—risk of CABG surgery according to the scale, PAD—peripheral arterial disease.

## Data Availability

The datasets used and/or analyzed during the current study are available from the corresponding author upon reasonable request.

## Supplementary Materials

The following supporting information can be downloaded at: https://www.mdpi.com/article/10.3390/jcm14020351/s1, Suppl. Table S1. Association of factors with the risk of developing MACE one year after coronary artery bypass grafting (Classification Table), Suppl. Table S2. Association of factors with the risk of developing MACE one year after coronary artery bypass grafting (Omnibus Tests of Model Coefficients), Suppl. Table S3. Association of factors with the risk of developing MACE one year after coronary artery bypass grafting (Model Summary).
