# Supplementary material for: Dynamics of Glycemic Status and Glucose Metabolism Markers 12 Months After Coronary Artery Bypass Grafting and Their Relationship with the Annual Prognosis of Patients
Source: J Clin Med. 2025 Jan 8;14(2):351. doi: 10.3390/jcm14020351 (PMC11765603; doi:10.3390/jcm14020351)
Supplement: Supplementary file 1 [file jcm-14-00351-s001.zip › jcm-3371308-supplementary.pdf]

Table S1. Association of factors with the risk of developing MACE one year after coronary artery bypass grafting (Classification Table)

			Predicted		
			MACE_year		
	Observed		1	2	Percentage Correct
Step 1	MACE_year	1	527	0	100.0
		2	28	0	0.0
		Overall Percentage			95.0
Step 2	MACE_year	1	527	0	100.0
		2	28	0	0.0
		Overall Percentage			95.0
Step 3	MACE_year	1	527	0	100.0
		2	28	0	0.0
		Overall Percentage			95.0
Step 4	MACE_year	1	527	0	100.0
		2	28	0	0.0
		Overall Percentage			95.0

Table S2. Association of factors with the risk of developing MACE one year after coronary artery bypass grafting (Omnibus Tests of Model Coefficients)

		Chi-square	df	Sig.
Step 1	Step	7.733	1	0.005
	Block	7.733	1	0.005
	Model	7.733	1	0.005
Step 2	Step	4.291	1	0.038
	Block	12.025	2	0.002
	Model	12.025	2	0.002
Step 3	Step	3.767	1	0.052
	Block	15.791	3	0.001
	Model	15.791	3	0.001
Step 4	Step	4.257	1	0.039
	Block	20.048	4	0.000
	Model	20.048	4	0.000

Table S3. Association of factors with the risk of developing MACE one year after coronary artery bypass grafting (Model Summary)

Step	-2 Log likelihood	Cox & Snell R Square	Nagelkerke R Square
1	214.089	0.014	0.042
2	209.797	0.021	0.065
3	206.031	0.028	0.085
4	201.774	0.035	0.108
